# Associations between High-Sensitivity C-Reactive Protein and Membrane Fluidity of Red Blood Cells in Hypertensive Elderly Men: An Electron Spin Resonance Study

**DOI:** 10.1155/2012/292803

**Published:** 2012-01-31

**Authors:** Kazushi Tsuda

**Affiliations:** ^1^Cardiovascular and Metabolic Research Center, Kansai University of Health Sciences, Senn-nann-gunn, Kumatori-cho, Wakaba 2-11-1, Osaka 590-0482, Japan; ^2^Division of Cardiology, School of Medicine, Wakayama Medical University, Wakayama 641-8509, Japan

## Abstract

Recent evidence indicates that high-sensitivity C-reactive protein (hs-CRP), an acute phase of an inflammatory marker, might be associated with atherosclerosis, hypertension, and other cardiovascular diseases. The present study was performed to assess the possible link between plasma hs-CRP and membrane fluidity (a reciprocal value of membrane microviscosity) in hypertensive elderly men. We measured the membrane fluidity of red blood cells (RBCs) in hypertensive and normotensive elderly men using an electron spin resonance and spin-labeling method. Membrane fluidity of RBCs was decreased in hypertensive elderly men compared with normotensive elderly men. Plasma hs-CRP levels were significantly higher in hypertensive elderly men than in normotensive elderly men. In contrast, plasma nitric-oxide- (NO-) metabolite levels were lower in hypertensive elderly men than in normotensive elderly men. The reduced membrane fluidity of RBCs was associated with increased plasma hs-CRP and decreased plasma NO-metabolite levels. In a multivariate regression analysis, plasma hs-CRP was an independent determinant of membrane fluidity of RBCs after adjustment for general risk factors. The results suggest that CRP might have a close correlation with the rheologic behavior of RBCs and the microcirculation and would contribute, at least in part, to the circulatory dysfunction and vascular complications in hypertensive elderly men.

## 1. Introduction

Evidence indicates that inflammation may actively participate in the development and progression of atherosclerosis and cardiovascular disease processes [1]. It is well recognized that high-sensitivity C-reactive protein (hs-CRP), an acute-phase of inflammatory marker, might be associated with increased risk of cardiovascular events [2, 3]. Recently, it has been shown that CRP could reduce the nitric oxide (NO) bioavailability by itself, which would induce endothelial and cardiovascular dysfunctions. Venugopal et al. demonstrated that CRP directly decreased endothelium type of NO synthase (eNOS) expression in human aortic endothelial cells in vitro [4]. Qamirani et al. showed that CRP inhibited endothelium-dependent NO-mediated dilatation of porcine coronary arterioles [5]. In a clinical study, it was also demonstrated that increased levels of hs-CRP were associated with reduced endothelium-mediated dilatory responses of the arteries [6]. However, the precise role of inflammation in the circulatory dysfunction in hypertension remains unclear. 

It has been proposed that abnormalities in physical properties of the cell membranes may underlie the defects that are strongly linked to hypertension, stroke, and other cardiovascular disease conditions [7–9]. An electron spin resonance (ESR) and spin-labeling method has been developed to evaluate the membrane fluidity (a reciprocal value of membrane microviscosity) and perturbations of the membrane function by external agents [8, 9]. The membrane fluidity is a physicochemical feature of biomembranes and is an important factor in modulating the cell rheologic behavior [8, 9]. Using the ESR method, we have been performing a series of experiments regarding the membrane fluidity of red blood cells (RBCs) in hypertension and have shown that membrane fluidity was significantly lower in hypertensive subjects than in normotensive subjects, particularly in the elderly [10–15]. Because the deformability of RBCs might be highly dependent on the membrane fluidity [8, 9], the reduction in membrane fluidity could cause a disturbance in the blood rheologic behavior and the microcirculation, which might contribute to the pathophysiology of hypertension and other circulatory disorders. In the present study, in order to assess the role of inflammation in the regulation of membrane function in hypertension in the elderly, we investigated the relationships between plasma hs-CRP and membrane fluidity of RBCs in hypertensive and normotensive elderly men using the ESR and the spin-labeling method.

## 2. Subjects and Methods

### 2.1. Subjects

A total of 29 men with untreated essential hypertension (age 63 ± 2 years old) were studied and compared with 18 age-matched normotensive men (age 64 ± 2 years old) (Table 1). The characteristics and laboratory findings in both groups were shown in Table 1. All subjects had no history of haematologic or hepatic disorders. All men were nonsmokers. They had similar life styles and dietary habits and were instructed to avoid any changes in dietary habits at least 12 weeks before the study. The study was approved by a local research committee of Kansai University of Health Sciences. Written informed consent was obtained from all participants when they were informed about the nature and objective of the study.

### 2.2. Electron Spin Resonance (ESR) Measurements of RBCs

**Figure 1:**
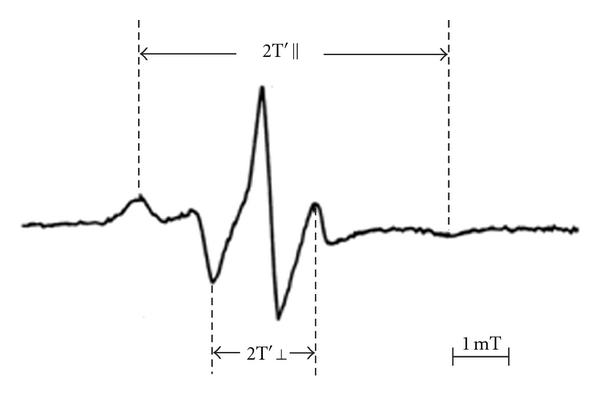
Representative electron spin resonance (ESR) spectrum of red blood cells (RBCs) for the fatty acid spin-label agents (5-nitroxide stearate). We calculated the order parameter (S) from outer and inner hyperfine splitting (2T′|| and 2T′⊥[10–16]). The greater the value of the order parameter (S) was, the lower the membrane fluidity of RBCs was [10–16]. (S: order parameter, 2T′||: outer hyperfine splitting, 2T′⊥: inner hyperfine splitting, T: tesla.)

Blood sampling was performed by venipuncture after 30 minutes of bed rest while fasting. The procedures of RBC preparation and ESR measurements were shown previously [9–15]. We evaluated the values of outer and inner hyperfine splitting (2T′|| and 2T′⊥ in tesla (T), resp.) in the ESR spectrum for the spin label agents (5-nitroxide stearate, Aldrich Co., Ltd., Milwaukee, WI, USA) (Figure 1), and calculated the order parameter (S) [10–16]. The greater the value of the order parameter (S) was, the lower the membrane fluidity of RBCs was. 

### 2.3. Nitric Oxide (NO) Metabolites (Nitrite and Nitrate) Analysis

The plasma levels of NO metabolites (nitrite and nitrate) were measured according to the method described previously [17].

### 2.4. Statistical Analysis

Values are expressed as mean ± SEM. The differences between hypertensive and normotensive men were analyzed using an unpaired Student's *t*-test. Linear regression analysis was performed to assess the relationships between membrane fluidity (order parameter: S) of RBCs and plasma hs-CRP or NO metabolite levels. Multivariate regression analysis with membrane fluidity (order parameter: S) of RBCs as a dependent variable and plasma hs-CRP, age, body mass index (BMI), hypercholesterolemia (more than 220 mg/dL), hyperglycemia (more than 110 mg/dL), and systolic blood pressure as independent variables was also performed. A *P*  value less than 0.05 was accepted as the level of significance. 

## 3. Results

### 3.1. Membrane Fluidity of RBCs in Hypertensive and Normotensive Elderly Men

The order parameter (S) for 5-nitroxide stearate in the ESR spectra of RBCs was significantly higher in hypertensive elderly men (HT) than in normotensive elderly men (NT) (HT 0.729 ± 0.002, mean ± SEM, *n* = 29, NT 0.718 ± 0.002, *n* = 18, *P* < 0.01). The finding indicated that membrane fluidity of RBCs was significantly lower in hypertensive elderly men than in normotensive elderly men.

### 3.2. Plasma High-Sensitivity C-Reactive Protein and Plasma Nitric-Oxide-Metabolite Levels in Hypertensive and Normotensive Elderly Men

**Figure 2:**
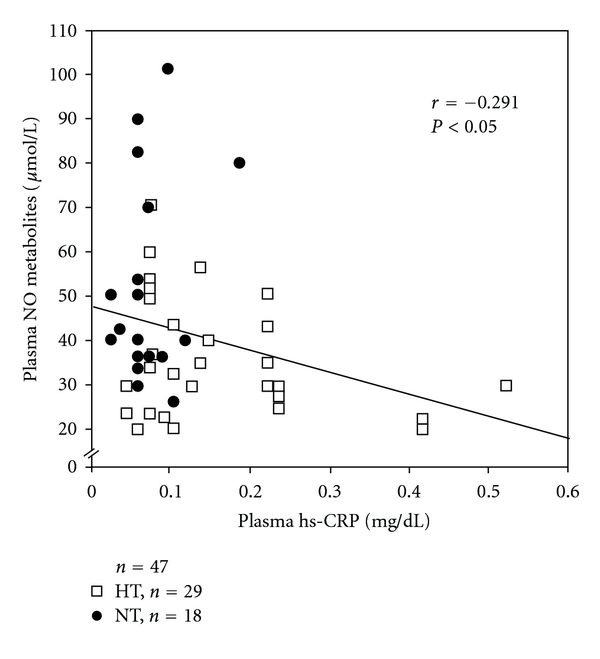
Inverse correlation between plasma high-sensitivity C-reactive protein (hs-CRP) and plasma nitric-oxide- (NO-) metabolite levels in hypertensive and normotensive elderly men.

The plasma hs-CRP levels were significantly higher in hypertensive elderly men than in normotensive elderly men (HT: 0.157 ± 0.022 mg/dL, *n* = 29, NT: 0.072 ± 0.009 mg/dL, *n* = 18, *P* < 0.01). In contrast, the plasma NO metabolites were lower in hypertensive elderly men than in normotensive elderly men (HT: 36.0 ± 2.4 *μ*mol/L, *n* = 29, NT: 52.5 ± 5.2 *μ* mol/L, *n* = 18, *P* < 0.01). In addition, in the overall analysis of hypertensive and normotensive elderly men, plasma hs-CRP levels were inversely correlated with plasma NO metabolites (*r* = − 0.291, *n* = 47, *P* < 0.05) (Figure 2). 

### 3.3. Relationship between Membrane Fluidity of Red Blood Cells and Plasma High-Sensitivity C-Reactive Protein, or Plasma Nitric-Oxide-Metabolite Levels in Hypertensive and Normotensive Elderly Men

**Figure 3:**
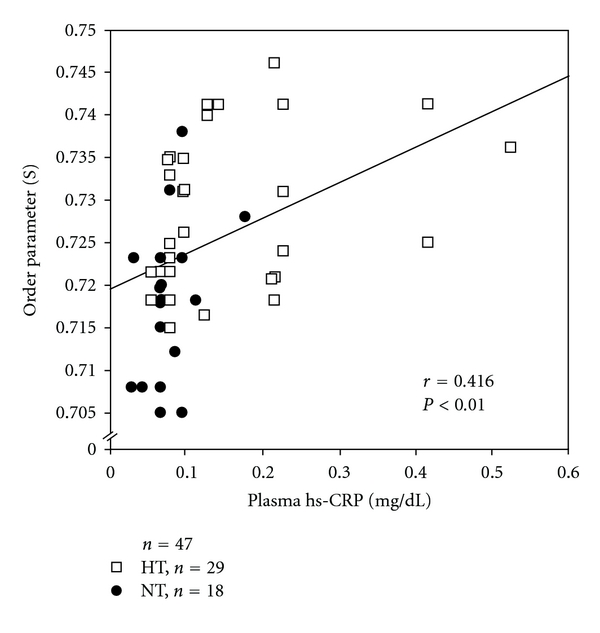
Correlation between plasma high-sensitivity C-reactive protein (hs-CRP) and membrane fluidity (order parameter: S) of red blood cells (RBCs) in hypertensive and normotensive elderly men.

The order parameter (S) of RBCs was significantly correlated with plasma hs-CRP levels (*r* = 0.416, *n* = 47, *P* < 0.01) (Figure 3) and was inversely correlated with plasma NO metabolite levels (*r* = −0.362, *n* = 47, *P* < 0.05). 

In a multivariate regression analysis after adjustment for general risk factors, plasma hs-CRP was an independent determinant of membrane fluidity (order parameter: S) of RBCs (Table 2).

## 4. Discussion

Evidence indicates that hs-CRP, an acute-phase of inflammatory marker, might be associated with increased risk of cardiovascular events [2, 3]. In the present study, we assessed the relationships between plasma hs-CRP levels and the membrane fluidity (a reciprocal value of membrane microviscosity) of RBCs in hypertensive and normotensive elderly men using the ESR and the spin-labeling method. The present study showed that the membrane fluidity of RBCs was decreased in hypertensive elderly men compared with normotensive elderly men. The result might be consistent with our previous findings showing that the cell membranes were stiffer and less fluid in hypertensive subjects [10–15]. Plasma hs-CRP levels were significantly higher in hypertensive elderly men than in normotensive elderly men and correlated with the order parameter (S) of RBCs, indicating that the reduced membrane fluidity of RBCs might be associated with elevated inflammatory status. To our knowledge, this is the first report demonstrating that CRP might have a close correlation with membrane fluidity of RBCs in humans. Multivariate regression analysis also showed that plasma hs-CRP was an independent determinant of membrane fluidity of RBCs after adjustment for general risk factors. Because the deformability of RBCs might be highly dependent on the membrane fluidity [8, 9], the reduction in membrane fluidity associated with increased hs-CRP levels could cause a disturbance in the blood rheologic behavior and the microcirculation.

It was shown that shear rate, shear stress, and blood viscosity were correlated with membrane fluidity of RBCs [18]. The finding proposed that in vivo shear forces might participate in the control of RBC membrane fluidity and that RBCs might adapt their membrane properties to blood flow conditions. It was also demonstrated that RBC membranes might become more rigid after myocardial infarction, which could contribute to the decreased RBC deformability and the increased blood viscosity in this group of patients [19]. On the other hand, Cazzola et al. [20] reported that the membrane fluidity of RBCs was decreased in the obese subjects and proposed that a decrease in RBC membrane fluidity could contribute to a reduction of the rate of blood flow and the oxygen diffusion through the RBC membranes and its exchange with tissues. It might be, therefore, possible that alterations in RBC membrane fluidity with elevated hs-CRP levels would be strongly linked to the progression of circulatory disorders.

Recently, it was demonstrated that CRP might directly impair the NOS expression in human aortic endothelial cells in vitro [4]. It was also shown that endothelium-dependent vasodilatory responses or microvascular endothelial functions were reduced in humans with elevated plasma hs-CRP levels [6, 21]. The results of the present study demonstrated that plasma hs-CRP levels were inversely correlated with plasma NO metabolites in the overall analysis of hypertensive and normotensive elderly men. One hypothesis is that higher hs-CRP levels could be accompanied by the reduced NO production and endothelial dysfunction. In a study presented earlier, it was shown that an NO donor significantly improved membrane fluidity of RBCs in hypertensive subjects, indicating that NO could have a beneficial effect on the rheologic behavior of RBCs and the microcirculation in hypertension [13–15]. We also demonstrated that the reduced membrane fluidity of RBCs was associated with the decreased plasma NO metabolites in overall analysis of hypertensive and normotensive elderly men, which might be consistent with our previous findings [13, 17]. It is, therefore, strongly suggested that the effects of CRP on membrane fluidity of RBCs might be mediated, at least in part, by the impaired NO bioavailability, although direct actions of CRP on membrane structural and functional properties cannot be excluded. Further studies should be performed to assess more precisely the relationships between CRP and NO and their role in the regulation of membrane functions and circulatory mechanisms in hypertension.

## 5. Conclusion

The results of the present study demonstrated that plasma hs-CRP levels were elevated in hypertensive elderly men compared with normotensive elderly men. In addition, it was shown that the reduced membrane fluidity of RBCs was correlated with higher plasma hs-CRP and lower plasma NO metabolite levels, indicating that abnormalities in RBC membranes might be associated with increased inflammatory status and endothelial dysfunction in hypertension. Although this is a cross-sectional and correlative study in Japanese men, the results of the present study suggest that CRP might have a close correlation with the rheologic behavior of RBCs and the microcirculation and would contribute, at least in part, to the circulatory dysfunctions and vascular complications in hypertensive elderly men. Moreover, a better knowledge of the inflammatory biomarker and cellular mechanisms underlying membrane abnormalities could provide useful information concerning the pathogenesis, treatment, and prognosis of hypertension in the elderly.

## Figures and Tables

**Table 1:** Clinical characteristics and laboratory findings of hypertensive (HT) and normotensive (NT) men.

	NT	HT
Number of subjects	18	29
Age (y.o.)	64 ± 2	63 ± 2
Body mass index (kg/m^2^)	24.2 ± 0.7	24.1 ± 0.5
Systolic blood pressure (mmHg)	124 ± 2	147 ± 1*
Diastolic blood pressure (mmHg)	69 ± 2	87 ± 1*
Heart rate (beats/min)	75 ± 2	72 ± 2
Erythrocyte counts (10^4^ cells/*μ*L)	458 ± 11	474 ± 8
Hemoglobin (g/dL)	14.2 ± 0.4	14.1 ± 0.2
Hematocrit (%)	43.2 ± 1.0	42.9 ± 0.6
Leucocyte counts (10^3^ cells/*μ*L)	5.5 ± 0.3	5.4 ± 0.2
Platelets (10^4^ cells/*μ*L)	21 ± 1	23 ± 1
Total cholesterol (mg/dL)	211 ± 6	209 ± 7
High-density lipoprotein cholesterol (mg/dL)	51 ± 2	52 ± 3
Low-density lipoprotein cholesterol (mg/dL)	134 ± 6	127 ± 7
Triglycerides (mg/dL)	120 ± 11	131 ± 12
Serum sodium (mmol/L)	140.8 ± 0.1	140.1 ± 0.2
Serum potassium (mmol/L)	4.0 ± 0.1	4.0 ± 0.1
Serum creatinine (mg/dL)	0.8 ± 0.1	0.9 ± 0.1
Fasting plasma glucose (mg/dL)	109 ± 3	116 ± 8

Values are mean ± SEM. *P < 0.05 between HT and NT.

**Table 2:** Multivariate regression analysis for predicting order parameter (S) of RBCs.

	SRC	*t*-value	*P* value
Age (y.o)	−0.215	−1.288	0.2053
Body mass index (kg/m^2^)	−0.197	−1.190	0.2409
Hypercholesterolemia (≧220 mg/dL)	−0.269	−1.986	0.0539
Hyperglycemia (≧110 mg/dL)	0.178	1.318	0.1951
Systolic blood pressure (mmHg)	0.339	2.552	0.0146
Plasma hs-CRP (mg/dL)	0.353	2.603	0.0129

*R* ^2^ = 0.373, *n* = 47, *F* = 3.972, *P* = 0.0033.

SRC: standard regression coefficient.
